# Transcriptional and Biochemical Characterization of Cytosolic Pyruvate Kinases in *Arabidopsis thaliana*

**DOI:** 10.3390/plants9030353

**Published:** 2020-03-11

**Authors:** Sabine Wulfert, Sören Schilasky, Stephan Krueger

**Affiliations:** Institute for Plant Sciences, University of Cologne, Zülpicherstraße 47b, 50674 Cologne, Germany; s.wulfert@mail.de (S.W.); soeren.schilasky@googlemail.com (S.S.)

**Keywords:** pyruvate kinase, glycolysis, respiratory metabolism

## Abstract

Glycolysis is a central catabolic pathway in every living organism with an essential role in carbohydrate breakdown and ATP synthesis, thereby providing pyruvate to the tricarboxylic acid cycle (TCA cycle). The cytosolic pyruvate kinase (cPK) represents a key glycolytic enzyme by catalyzing phosphate transfer from phosphoenolpyruvate (PEP) to ADP for the synthesis of ATP. Besides its important functions in cellular energy homeostasis, the activity of cytosolic pyruvate kinase underlies tight regulation, for instance by allosteric effectors, that impact stability of its quaternary structure. We determined five cytosol-localized pyruvate kinases, out of the fourteen putative pyruvate kinase genes encoded by the *Arabidopsis thaliana* genome, by investigation of phylogeny and localization of yellow fluorescent protein (YFP) fusion proteins. Analysis of promoter β-glucuronidase (GUS) reporter lines revealed an isoform-specific expression pattern for the five enzymes, subject to plant tissue and developmental stage. Investigation of the heterologously expressed and purified cytosolic pyruvate kinases revealed that these enzymes are differentially regulated by metabolites, such as citrate, fructose-1,6-bisphosphate (FBP) and ATP. In addition, measured in vitro enzyme activities suggest that pyruvate kinase subunit complexes consisting of cPK2/3 and cPK4/5 isoforms, respectively, bear regulatory properties. In summary, our study indicates that the five identified cytosolic pyruvate kinase isoforms adjust the carbohydrate flux through the glycolytic pathway in *Arabidopsis thaliana*, by distinct regulatory qualities, such as individual expression pattern as well as dissimilar responsiveness to allosteric effectors and enzyme subgroup association.

## 1. Introduction

During plant development and adaptation to environmental changes, the glycolytic network provides an enormous metabolic flexibility. Thereby, flux regulation is achieved by the fine control of key regulatory enzymes, including pyruvate kinase (PK). PK-mediated synthesis of pyruvate represents a bottleneck for acetyl-CoA entering the TCA cycle. On the other hand, reduced pyruvate kinase activity will lead to a backlog of PEP and other glycolytic intermediates, thereby increasing the flux rate of carbon skeletons into branching biosynthetic pathways. The *Arabidopsis thaliana* genome encodes several putative cytosolic and plastidial PKs, and glycolytic metabolites can be exchanged between the cytosol and plastids [1] since both compartments are connected through diverse transporters located in the inner plastid envelope membrane [2,3,4]. Despite the assumable key regulatory function of pyruvate kinase so far, only plastidial isoforms have been described [5]. Possible reasons for this are numerous. The high number of isoenzymes with potential redundant physiological roles, as well as the compartmentalized system with glycolytic intermediates equilibrating through plastid membrane transporters, may hamper their investigation.

Several ways of regulation for PKs have been verified, including binding of co-substrates and allosteric effectors. In *Saccharomyces cerevisiae* the glycolytic intermediate fructose-1,6-bisphosphate (FBP) increases the affinity to the bivalent cations, Mg^2+^ or Mn^2+^, which are essential for PK activity [6]. Studies on cytosolic PK from castor bean identified glutamate as the most effective inhibitor, whereas aspartate functioned as an activator [7]. Furthermore, the TCA cycle intermediates citrate, 2-oxoglutarate, fumarate and malate have the potential to decrease activity of some plant PKs, which indicates a role as feedback regulators [8,9,10]. A further regulatory aspect may arise from pH-dependent alterations in the PK enzyme’s affinity to metabolite inhibitors. This was proposed in a study on PK enzymes that were isolated from cotyledons of *Ricinus communis* [8]. An enhanced PK activity was accompanied by a reduced cytosolic pH, which was caused by H^+^-symport that affected the uptake of endosperm-derived sugars and amino acids. Protein degradation is another way of controlling PK activity as shown recently for cotton cytosolic pyruvate kinase 6 (GhPK6) [11]. Here phosphorylation-mediated ubiquitination of GhPK6 appears to modulate the cotton fiber elongation process. Subunit association/dissociation was shown to be an additional mechanism to adjust PK activity. Accordingly, human pyruvate kinase muscle isoenzyme 2 (PKM2) assembled to a dimer has remarkably lower affinity to PEP as the respective tetramer [12]. This regulatory mechanism has been proposed for plant PKs as well, as in vitro studies show that specific subgroup combinations are more active than others [5,7]. Plastidial PKs in *Arabidopsis thaliana* have been shown to be essential for seed oil production, whereby enzyme isoforms form higher-order subunit complexes composed of 4*α* and 4*β*-subunits [5].

Spatial distribution of glycolytic enzymes within the cell constitutes a further point of regulation, since enzymes may localize at sites of demand for glycolytic intermediates. Proteomic analyses of highly purified mitochondrial fractions revealed the presence of glycolytic key enzymes, including PK isoforms on the outside of the mitochondrion considered to ensure direct import of pyruvate [13]. Furthermore, degradation by the proteasome determines PK-catalyzed pyruvate synthesis, since C-terminal proteolytic processing of cytosolic PK was shown for isoforms derived from soybean [14]. Finally, the multilayered character of PK activity control generates a complex picture on its role as a flux regulator. On the other hand, the complexity of the involved factors underlines the enormous sensitivity by which fine control is attained.

The *Arabidopsis thaliana* genome encodes 14 putative pyruvate kinases, which are likely to be isoforms catalyzing the ADP-dependent conversion of PEP to pyruvate, thereby releasing ATP. These isoenzymes show a broad diversity concerning gene expression rate and tissue specificity [15] and additionally segregate into plastidial and cytosolic subclades according to consensus predictions of their subcellular localization [16].

We identified five cytosolic PK gene candidates that show a significant expression and are likely to be localized to the cytosol. After having confirmed the cytosolic localization of PK2, PK4 and PK5 by heterologous expression of yellow fluorescent protein (YFP) fusion constructs in *Nicotiana benthamiana*, we aimed to identify the different roles of cytosolic PK enzymes in dependence of changing developmental and environmental conditions. By histochemical analysis of plant lines expressing promoter-β-glucuronidase (GUS) fusion constructs, we observed tissue-specific localization of PK expression in diverse developmental stages. Furthermore, biochemical characterization of purified cPK isoenzymes heterologously expressed in *Escherichia coli* showed that PK activity is controlled by the presence of metabolite effectors or binding of enzyme subunits.

In summary, our findings show that regulation of cPK enzyme activity is controlled by distinct gene expression patterns, different sensitivity to allosteric effectors and enzyme subgroup formation.

## 2. Results

### 2.1. Selection of Pyruvate Kinase Candidates to be Involved in Cytosolic Glycolysis

**Figure 1:**
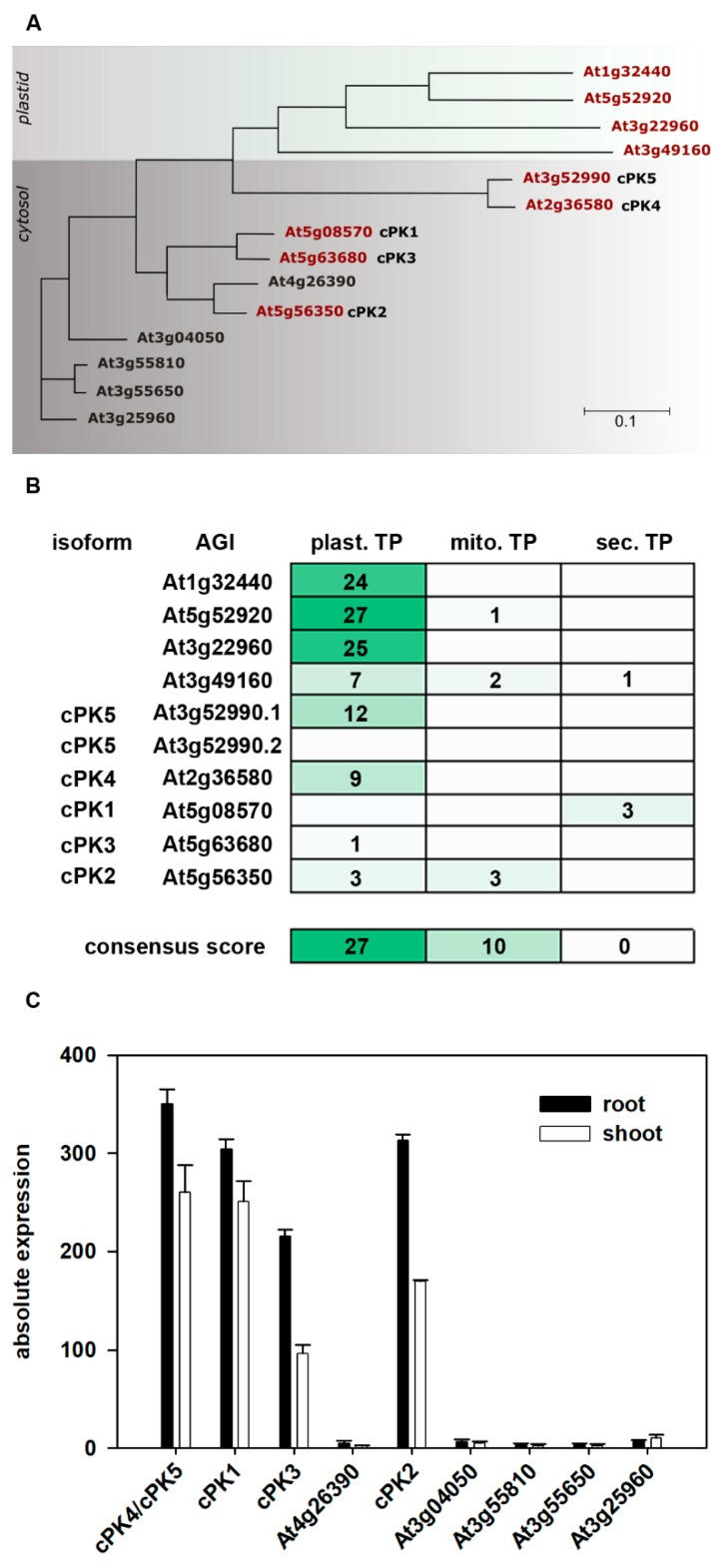
(**A**) Dendrogram of the *Arabidopsis thaliana* pyruvate kinase family based on protein sequences obtained from the Aramemnon database [16]. The alignment was performed using clustalW, and the dendrogram was created using Dendrocsope 3 [17]. The scale bar represents the number of substitutions per site. Significantly expressed genes are highlighted in red (according to the *Arabidopsis* efp browser). (**B**) Consensus prediction of the subcellular location. Consensus scores for PK proteins obtained from the Aramemnon database [16]. (**C**) Expression of putative cytosolic *PK* genes in roots and leaves. Data obtained from published microarray-data, *Arabidopsis* efp browser [15].

**Figure A1:**
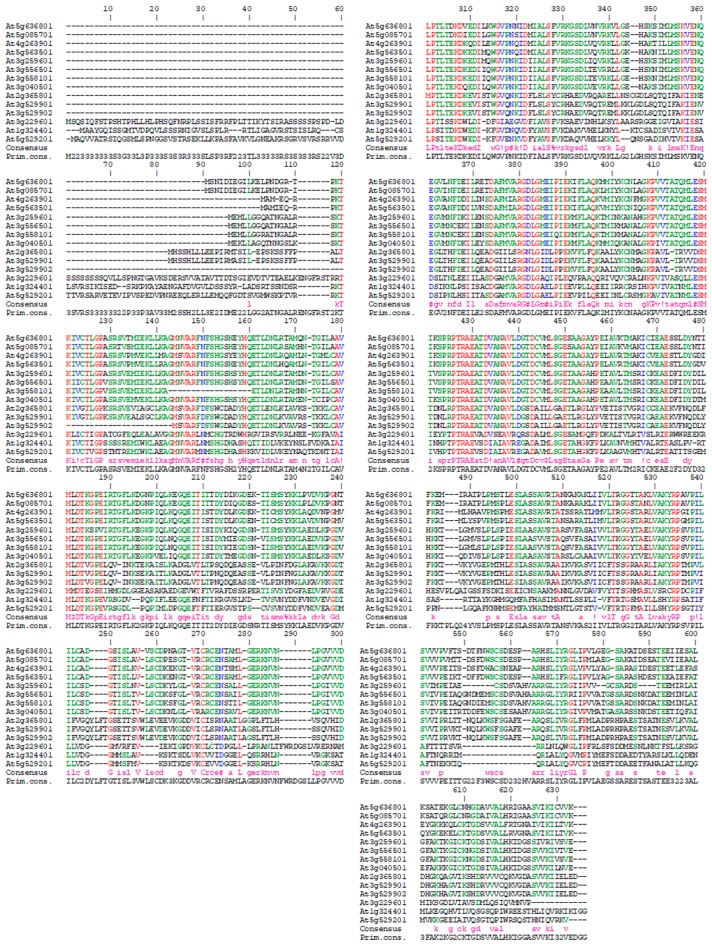
Multiple sequence alignment of *Arabidopsis thaliana* pyruvate kinases.

**Figure A2:**
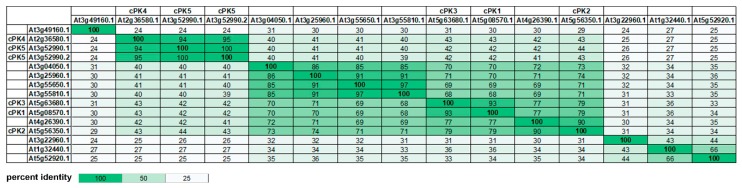
Percentages of identity between *Arabidopsis thaliana* PK isoenzymes based on multiple sequence alignments of the respective amino acid sequences. The percentage of identity is shown in numbers and visualized by a green color gradient.

The *Arabidopsis thaliana* genome encodes for 14 putative PK isoforms. A phylogenetic tree based on PK amino acid sequence alignment (Appendix Figure A1 and Figure A2) is shown in Figure 1A. Four PK isoforms, namely At1g32440, At5g52920, At3g22960 and At3g49160, are predicted to contain a chloroplast transit peptide according to the Aramemnon database [16], and their localization to the chloroplast was confirmed in vitro by Andre and colleagues (2007) [5]. For the remaining isoforms, the consensus predictions give no clear indication for targeting to a certain organelle (Figure 1B). An alignment of protein sequences of the *Arabidopsis* PK candidates with bona fide PKs from other organisms revealed two PK subclades to exist in *Arabidopsis* [5]. In addition to isoforms localized to the plastids, another subclade consisting of enzymes that target the cytosol was hypothesized.

**Figure 2:**
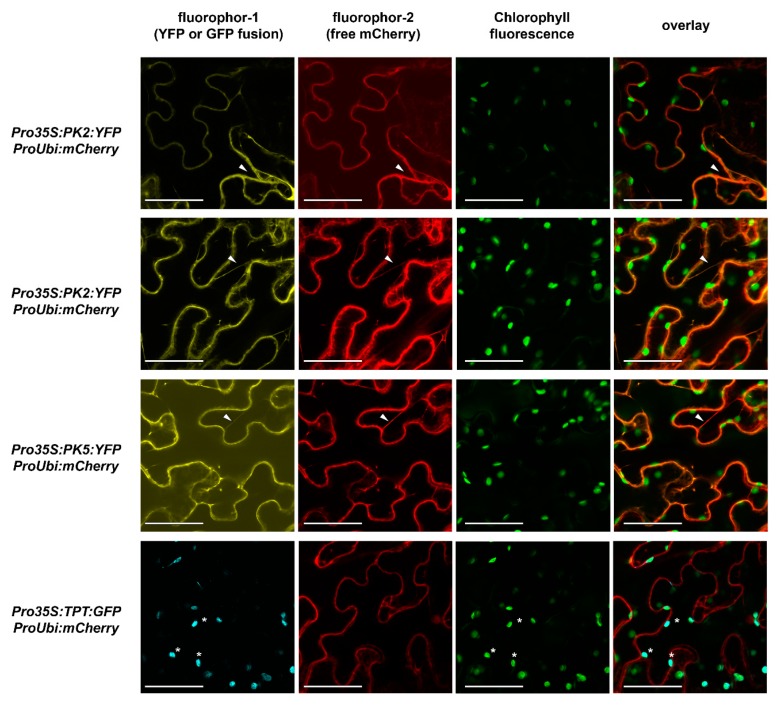
Subcellular localization of pyruvate kinase isoforms. cPK2, cPK4, cPK5-YFP fusion proteins (yellow) and TPT-GFP fusion protein (cyan) were expressed under the control of the cauliflower mosaic virus promoter (*Pro35S*), while free mCherry fluorescence protein was expressed under the control of the ubiquitin promoter (*ProUbi*). All PK-YFP fusion proteins were co-localized with free mCherry in cytosolic plasma strands (white arrows), whereby TPT-GFP fusion protein was co-localized with chlorophyll A fluorescence (green) as indicated by the white asterisks. Constructs were transiently expressed in *Nicotiana benthamiana* leaves. Scale bars = 50 µm.

According to the *Arabidopsis* efp browser expression database [15], only five out of ten candidate genes coding for putative cytosolic PKs are expressed up to a reasonable level in order to be considered for our analysis (Figure 1C). Relative expression of these putative cytosolic isoforms is generally higher in roots than in leaves as indicated by published microarray data (Figure 1C). Expressed putative cytosolic pyruvate kinase (cPK) candidates were selected for further analysis and are subject of the current study. For clear determination, the genes were named as follows: At5g08570, *cPK1*; At5g56350, *cPK2*; At5g56350, *cPK3*; At2g36580, *cPK4;* and At3g52990, *cPK5*. PK4 and PK5 expression cannot be distinguished from each other, as both genes are identified by the same probe target. Based on an alignment with bona fide PKs from other organisms, the five expressed PK candidates fall into a subclade of cytosol localized isoforms [5]. As for cPK2, cPK4 and cPK5, the localization prediction was unclear, and YFP fusion proteins were constructed for these enzymes and transiently co-expressed with free mCherry fluorescence protein in leaf epidermal cells of *Nicotiana benthamiana*. Confocal laser scanning microscopy analysis of transformed leaf sections revealed that, in contrast to the triose-phosphate/phosphate translocator (TPT) green fluorescent protein (GFP) fusion, all cPK:YFP fusion proteins were co-localized with free mCherry protein in the cytosol and were absent from the chloroplast (Figure 2).

### 2.2. Pyruvate Kinase Genes Show an Isoform-Specific Expression Pattern

**Figure 3:**
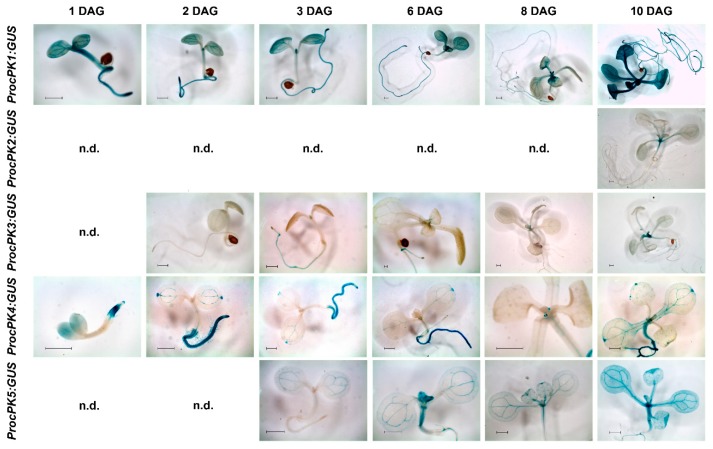
Histochemical β-glucuronidase (GUS) localization in seedlings of the *cPK1*, *cPK2*, *cPK3*, *cPK4* and *cPK5* promoter-GUS constructs, one day after germination (DAG) up to ten DAG. Representative images of one out of three individual transgenic lines are shown. n.d. indicates developmental stages without detectable GUS activity. Bars indicate 0.5 mm.

**Figure 4:**
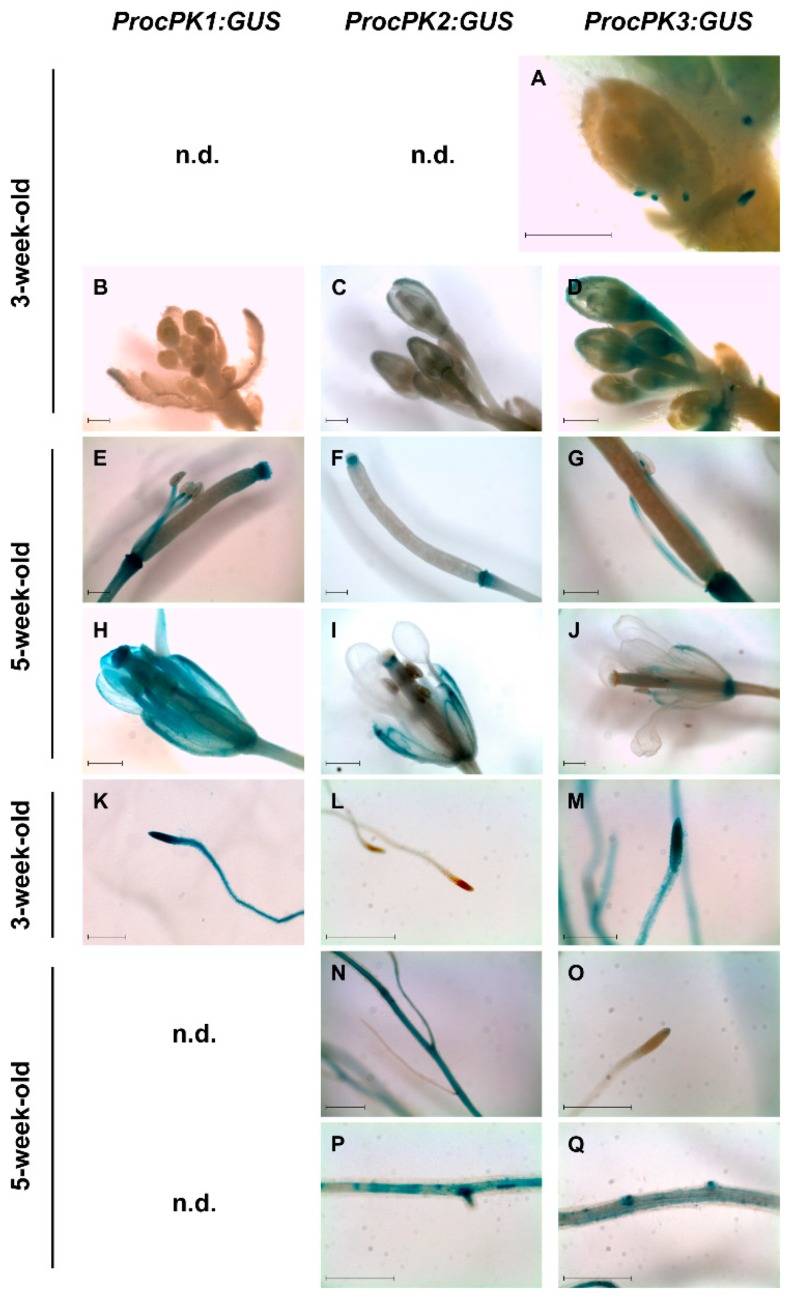
Histochemical GUS localization in flower organs (**A**–**G**) and in roots (**K**–**Q**), for the *cPK1*-promoter-GUS (**B**,**E**,**H**,**K**), the *cPK2*-promoter-GUS (**C**,**F**,**I**,**L**,**N**,**P**) and the *cPK3*-promoter-GUS (**A**,**D**,**G**,**J**,**M**,**O**,**Q**) in 3-week-old plants (**A**–**D**) and 5-week-old plants (**E**–**J**), is illustrated in developing flowers (**B**–**D**) and in fully developed carpels (**E**–**G**) with stamen as well as entire flowers (**H**–**J**) of 5-week-old plants. Pictures show root tips of 3-week-old plants (**K**–**M**) and GUS expression in 5-week-old plants, restricted to primary roots for the *cPK2*-promoter-GUS (**N**) and stem cells in root tips for the *cPK3*-promoter-GUS (**O**). Expression at sites of emerging secondary roots for the *cPK2*- and *cPK3*-promoter-GUS (**P**,**Q**). Representative images of one out of three different transgenic lines are shown. n.d. indicates plant organs without detectable GUS activity. Bars indicate 0.5 mm.

**Figure A3:**
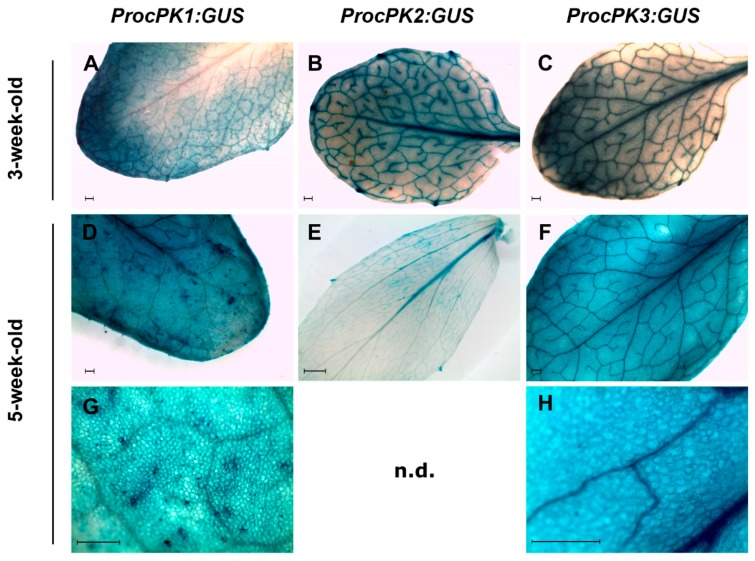
Histochemical GUS localization in oldest rosette leaves for the *cPK1* promoter (**A**,**D**,**G**), the *cPK2* promoter (**B**,**E**) and the *cPK3* promoter (**C**,**F**,**H**) in 3-week-old (**A**–**C**) and 5-week- old plants (**D**–**H**). Bars indicate 0.5 mm. G and H are magnifications of D and F, respectively. Representative images of one out of three different transgenic lines are shown.

The high number of expressed cytosolic PK isoforms allows each gene candidate to potentially fulfill distinct tasks during plant glycolysis. To assess tissue-specific expression of respective cPK genes in dependence on the developmental status of the plant, a β-glucuronidase (GUS) reporter approach was taken. Promoters of the five *cPK* candidates were fused to GUS and expressed in *Arabidopsis thaliana*. Subsequently, the plant organs were stained and analyzed in detail. The GUS-staining results suggest that the cytosolic isogenes *cPK1, 2* and *3* are expressed in vegetative and reproductive tissues, as promoter-GUS activity was observed in leaves, roots and flowers of plants from diverse developmental stages (Figure 3, Figure 4, Appendix Figure A3). However, for cPK4 and cPK5 a quite strong GUS signal was observed during seedling stage, and the same lines showed no or very low expression in tissues of mature plants under standard growth conditions.

Analysis of the first ten days of seedling development revealed clear differences in the expression of selected *cPK* isogenes (Figure 3). While the *cPK1* promoter lead to broadly abundant GUS activity in roots and leaves of seedlings from the first day on, respective lines for the *cPK2* and *cPK3* promoters showed comparable GUS signals only at later stages. Notably, *cPK1* promoter GUS lines displayed strong staining in the shoot apical meristem (Figure 3) and in the basal part of young leaves (Figure 2 promcPK1::GUS, 8 DAG). *cPK2* promoter GUS expression did not initiate in the rosette axis until ten days after germination. The *cPK3* promoter-induced expression was restricted to the root tip and was already visible on the first day after germination. From the third day on, the signal became abundant in the entire root and was also observed in the leaf axis and spatial related trichomes. In *cPK4*-promoter plants expression was observed from the first day on in the entire root and the leaf vasculature, and later in hydathodes and at the sides of emerging leaf buds (Figure 3, promcPK4::GUS, 8 DAG). In contrast, expression of *cPK5* started later and was mainly restricted to the cotyledon, leaf and root vasculature. As indicated before, promoter-GUS constructs for *cPK1*, *2* and *3* were expressed in the vasculature and mesophyll of rosette leaves with varying intensity depending on developmental stage of the leaf (Appendix, Figure A3). In roots, *cPK1* appeared to be ubiquitously expressed, whereas GUS activity for *cPK2* and *cPK3* promoters was limited to confined areas. The *cPK2* promoter GUS lines showed blue stains restricted to primary roots (Figure 4N), while the *cPK3* promoter primarily lead to GUS activity in younger roots and root tips (Figure 4M). Promoters of both *cPK2* and *cPK3* drive GUS expression at sites of emerging lateral roots (Figure 4P,Q). In young flowers of *cPK1* promoter-GUS lines, no signal was observed, whereas fully developed flowers appeared to be ubiquitously stained in sepals, petals, filaments of stamen, style and the stigma tissue (Figure 4B,E,H). *cPK2* was also not expressed in young flowers, whereas in later stages, GUS-staining was limited to sepals and style (Figure 4C,F,I). In contrast, *cPK3* promoter-dependent GUS expression was already detected in the abscission zones of young flower buds (A), and later expression became apparent in the anthers as well (G).

### 2.3. Cytosolic Pyruvate Kinases Respond to Cold Stress

**Figure 5:**
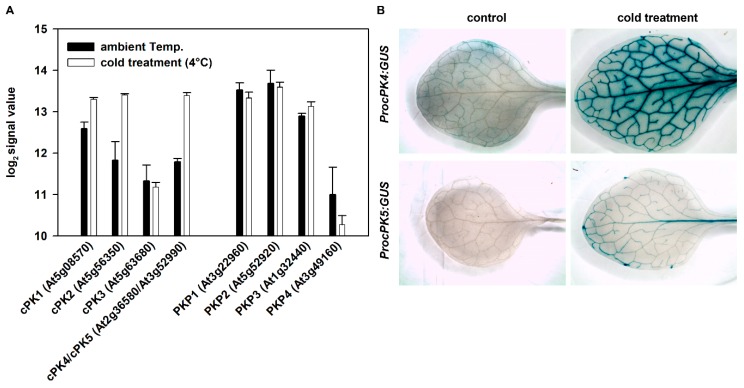
(**A**) Expression data of cytosolic and plastidic pyruvate kinases after cold treatment obtained from publicly available data source (https://genevestigator.com). Sixteen-day-old plants were transferred to the cold room (4 °C), and plant tissues were harvested at 24 h after onset of treatment. (**B**) To validate cold-induced expression of *cPK4* and *cPK5*, twenty-day-old *cPK4* and *cPK5* promoter GUS plants grown on soil were transferred to the cold room (4 °C) for three days. Control plants were grown under normal growth conditions. The oldest leaves of control and cold-treated plants were sampled, and the GUS activity was determined by histological staining. Representative images of one out of three different transgenic lines are shown.

In contrast to the plastid-localized pyruvate kinases, the analysis of public available transcript data [15] revealed that the cytosolic PKs are induced in response to cold treatment (Figure 4A). According to these data, *cPK1* and *cPK2* expression is induced, whereas *cPK3* transcript levels are not altered. The strongest induction upon cold treatment was found for the isogenes *cPK4* and *cPK5*. However, regarding the microarray data, *cPK4* and *cPK5* expression cannot be considered separately, since respective data do not discriminate between both genes. Thus, we validated these data by using *Arabidopsis* lines carrying the respective GUS-promoter constructs that were sampled after cold treatment (Figure 5B). In *cPK4*-lines, the GUS signal was very low in untreated control plants, and it was strongly induced in the leaf vasculature of cold treated plants. *cPK5* expression appeared to also be increased; however, the observed effect was not as strong as in the *cPK4*-GUS line. Therefore, our results indicate that cytosolic pyruvate kinases are induced during cold treatment.

### 2.4. Pyruvate Kinase Expression Follows a Diurnal Course

**Figure 6:**
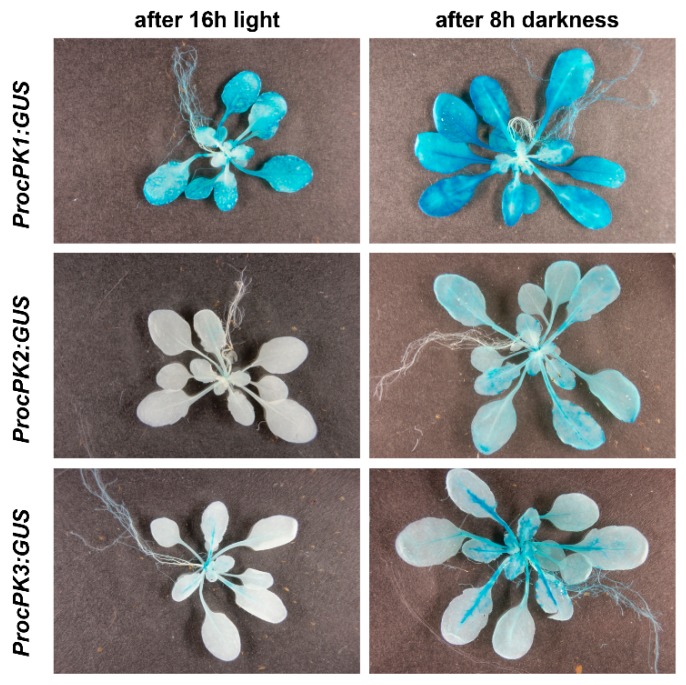
Histochemical GUS expression in dependence on the daytime in 4-week-old plants expressing the *cPK1*-promoter-GUS, *cPK2*-promoter-GUS and *cPK3*-promoter-GUS constructs, grown on soil sampled after 16 h light and after 8 h darkness. Representative images of one out of three different transgenic lines are shown.

Carbon metabolism is exposed to diurnal changes, since photosynthesis cannot maintain energy provision during the dark period. Breakdown of starch and soluble sugars during the night leads to increased activity of glycolytic enzymes. In order to assess cPK activity, which is a key enzyme in glycolysis, in the course of the day/night cycle, *cPK* promoter GUS plants were sampled at the end of the night (after 8 h darkness) and at the end of the day (after 16 h light). Apart from differences in signal intensity between the lines, *cPK1*, *cPK2* and *cPK3* promoters lead to increased GUS activity when sampled after 8 h of darkness compared to plants sampled after 16 h in the light (Figure 6). In contrast, *cPK4* and *cPK5* promoter-GUS lines showed no detectable GUS activity in mature plants used for the day/night cycle experiment (data not shown).

### 2.5. Kinetic Characterization of Cytosolic Pyruvate Kinases

**Figure A4:**
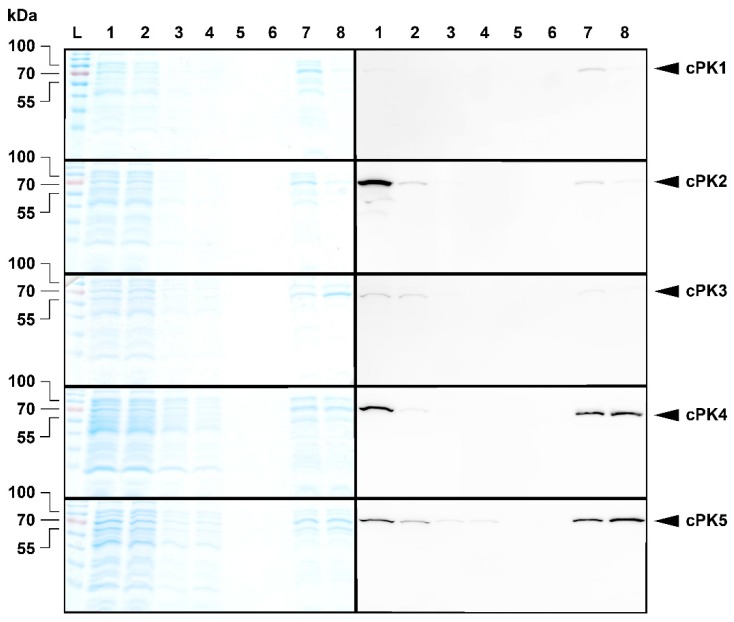
Affinity purification of heterologous expressed cytosolic pyruvate kinases. Protein extracts (2 µL) of different fractions (1, cell extract; 2, flow through; 3, wash fraction I; 4, wash fraction II; 5-8, elution fraction I-IV) were separated on SDS-polyacrylamide gels and stained either by Coomassie protein stain or blotted and detected by Western blot analysis using anti-His antibodies.

**Figure A5:**
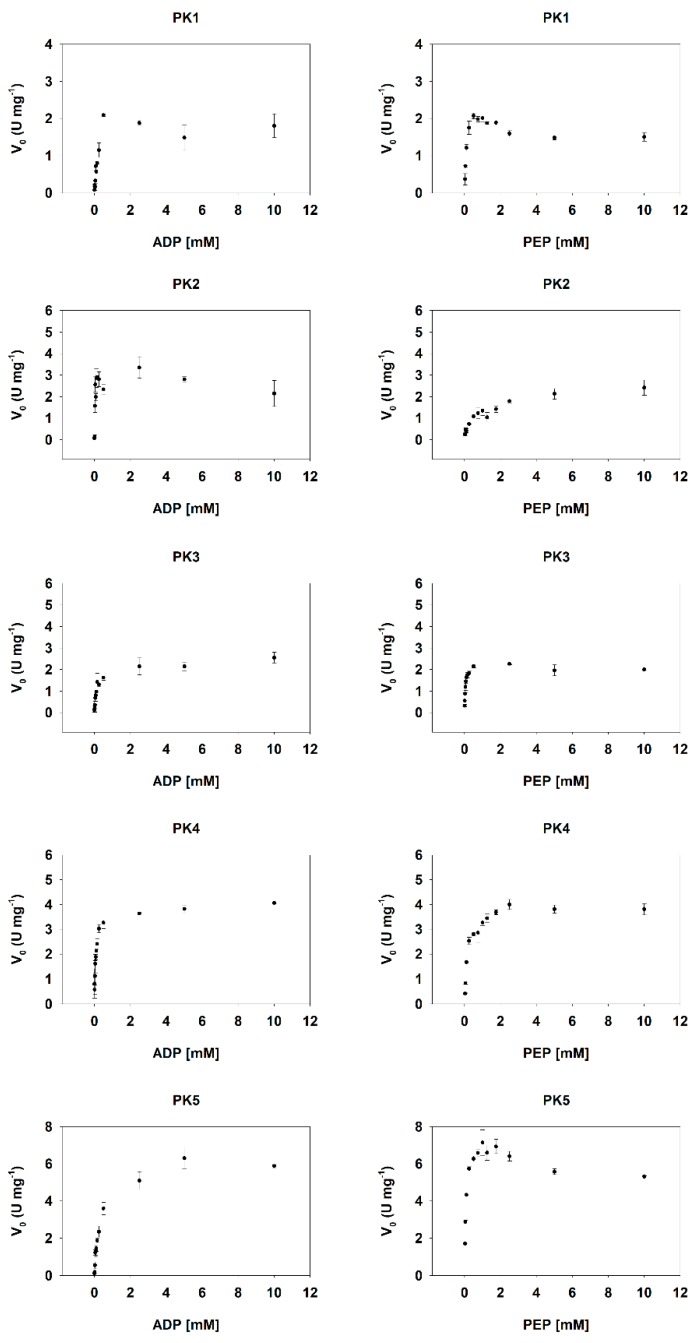
Biochemical characterization of cytosolic pyruvate kinase isoenzymes. Km values of cPK1, cPK2, cPK3, cPK4 and cPK5 for ADP (left panel) and PEP (right panel) were determined by nonlinear regression based on the Michaelis–Menten equation. Linear dilutions of ADP (0.005, 0.01, 0.025, 0.05, 0.075, 0.1, 0.15, 0.25, 0.5, 2.5, 5.0 and 10.0 mM) and PEP (0.025, 0.05, 0.1, 0.25, 0.5 0, 0.75, 1.0, 1.25, 1.75, 2.5, 5.0 and 10.0 mM) were used. Data are mean ± SD of four replicates.

To assess the kinetic parameters of cytosolic PK enzymes, coding sequences were cloned into the pET16B expression vector mediating N-terminal 6x-His tag. PK isoenzymes were expressed heterologously in *E. coli* and purified in order to obtain a sufficient yield (Appendix Figure A4). Enriched isoenzymes were characterized in vitro applying a lactate dehydrogenase coupled enzyme assay according to [18]. Plots showing cPK activity versus linear dilution series of ADP and PEP describe hyperbolic saturation curves; thus, biochemical properties like Michaelis constant *Km*, enzymes maximum rate *Vmax*, and turnover number *kcat* were calculated applying the Michaelis–Menten equation (Table 1; Appendix Figure A5). The lowest specific activity was observed for cPK1 with a *Vmax* of 1.4U/mg. On the contrary, PK5 exhibited the highest specific activity (*Vmax*: 6.4 U/mg). The *Km* values for ADP were in about the same range varying between 0.07 mM (cPK4) and 0.34 mM (cPK5). cPK2 had a quite high *Km* for PEP (0.76 mM), compared to the other isoenzymes.

### 2.6. Allosteric Effects on Pyruvate Kinase Enzyme Activity

PK enzymes are tightly regulated by allosteric effectors as known from other organisms [6,8,18]. To assess putative regulatory impact of diverse selected metabolites on cytosolic PK enzymes from *Arabidopsis thaliana*, single enzyme extracts were tested under substrate saturating conditions. ATP showed a strong effect on cPK activity. In the case of cPK1, the specific activity was reduced by more than 90%, and for cPK3 a reduction of 73% was observed (Table 2). In contrast, ATP did not affect the specific activity of cPK5. Our data indicate that cPK1 can be positively affected by FBP, whereby glutamate and aspartate lead to a decrease in specific enzyme activity. Activities of other cPK isoenzymes were not significantly altered by the selected metabolites. Citrate is a central metabolite of the citrate cycle and has been applied as negative control in several previous kinetic analyses of pyruvate kinases [10]. Also, in our study citrate had a strong negative allosteric effect on all five isoforms.

### 2.7. Effects of Subgroup Complex Formation

**Figure 7:**
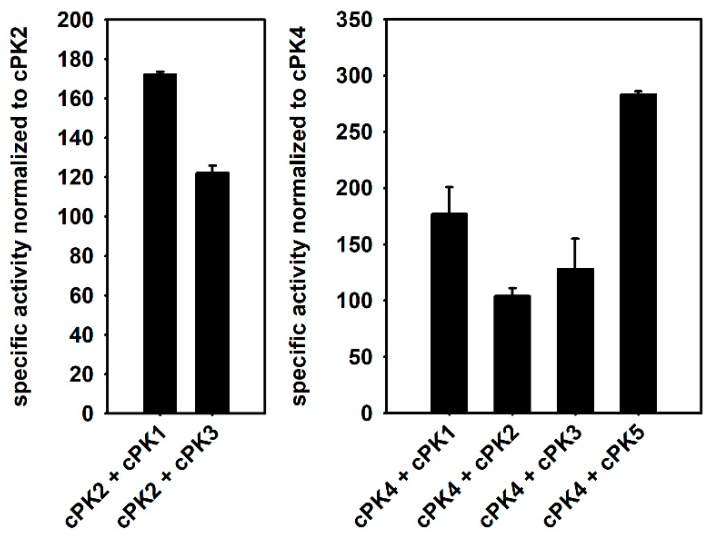
Effect of isoenzyme combinations on specific activities (U mg_protein_^−1^) of enzyme extracts containing equal amounts of two isoenzymes normalized to activity of extracts containing a single isoenzyme. Specific activity of single enzyme extracts of cPK2 and cPK4 were set to 100%.

It was shown for plastidial PKs from *Arabidopsis thaliana* that their activity is dependent on the formation of *α*- and *β*-subunits. This is based on the observation that in vitro catalytic efficiency is significantly higher for reconstituted subunit complexes in comparison to the respective subunit alone [5]. To test whether subgroup complex association also has an effect on the cytosolic isoenzymes, mixtures of equal ratios of cPK extracts were used for further kinetic experiments. Under substrate saturating conditions, specific activities were determined for mixtures of selected isoform pairs. In a mixture of cPK1 and cPK2, specific activity was strongly increased by about 70% compared to cPK2 alone (Figure 7). A positive effect on activity in the same range was observed in a mixture of cPK1 and cPK4 (75%). Combinations of cPK2 and cPK3 or cPK4 and cPK3 also increased specific activity compared to the single enzymes but only by about 20%. However, the highest impact was observed in a batch containing equal ratios of cPK4 and cPK5, here the activity was 180% higher than the activity of cPK4 alone.

## 3. Discussion

### 3.1. Arabidopsis thaliana Five Cytosolic Pyruvate Kinases Form Two Functional Subgroups

Various previous studies showed that plant PKs either localize to the cytosol or the plastid, whereby the subcellular distribution is of crucial relevance for enzyme activity. Plastidial PK isoforms are reported to be essential for biosynthesis of seed oil and the mobilization of storage compounds in *Arabidopsis thaliana* seedlings [5,19]. Cytosolic PKs on the contrary take over an important role in carbohydrate breakdown, cytosolic ATP biosynthesis and provision of pyruvate to the TCA cycle, as known from other plant and non-plant organisms [20]. Apart from their fundamental role in primary metabolism, reports on *Arabidopsis thaliana* PKs are limited to seed-expressed, plastid-localized isoforms [5]. In the present study, five potential cytosol-targeted PKs from *Arabidopsis thaliana* were identified based on phylogenetic studies and gene expression data [5] (Figure 1A). The prediction of cytosolic localization of cPK2 (At5g56350), cPK4 (At2g36580) and cPK5 (At3g52990) was confirmed by investigating YFP-fusion proteins in *Nicotiana benthamiana* leaf cells (Figure 2). Furthermore, PK activity was verified in vitro for all five isoforms since the isoenzymes were capable of hydrolyzing PEP into pyruvate and ATP (Table 1). Our observations suggest that within the subclade of cytosolic PKs, *cPK1*, *cPK2* and *cPK3* form a subgroup distinct from *cPK4* and *cPK5*, also displayed by structural characteristics of the genetic sequence. The specific roles for each subgroup were illustrated by GUS expression data, which showed entirely independent expression patterns for each isoform. Furthermore, the analysis of in vitro enzyme activity suggests regulatory properties of subunit complexes composed of cPK1/2, cPK2/3 or cPK4/5 isoforms, respectively (Figure 7).

### 3.2. Pyruvate Kinase Enzymes are Localized to the Cytosol

Three out of five PK candidates were localized to the cytosol via confocal microscopy of YFP-fusion proteins (Figure 2). This is in agreement with predictions based on a bona fide alignment of PK proteins from other plants [5]. Since consensus sequence predictions for cPK1 and cPK3 identified no target peptide for a specific organelle, we presume that both PKs also target to the cytosol [16]. Furthermore, formation of complexes with cPK2, cPK4 and cPK5 bears regulatory property of PK activity, assuming both isoforms of an enzyme pair to target to the same compartment.

### 3.3. Cytosolic Pyruvate Kinase Genes are Expressed in a Tissue-Specific and Developmental-Specific Manner

Our GUS studies show a distinct tissue-specific expression of the five *cPK* isogenes, also supported by publicly available microarray data [15]. Considering the fact that the cPK isoforms underlie distinct metabolic control, it can be concluded that plants are able to fine-tune cell and tissue-specific metabolism by coordinated *cPK* expression, in order to closely match the energy requirements under different conditions.

Cytosolic *PK1* appeared to be ubiquitously expressed in all tissues and in all developmental stages. This stands in contrast to *cPK2* and *cPK3* expression, which started at a later stage of seedling development and was initially restricted to meristematic tissues, such as the root tips (Figure 3) and to the proliferation zone of young leaves. However, all three isoforms were expressed in later developmental stages. In contrast, *cPK4* expression was pronounced in roots and cotyledons during the first days of development (Figure 3). At a later stage, *cPK4* together with *cPK5* was expressed only under stress conditions (Figure 5). Seedling establishment might be considered as a stress situation, since the plants face limited carbon supply because of an inactive or not yet fully activated photosynthetic machinery. Accordingly, during this stage no carbon assimilation takes place, and the plants depend on the degradation of compounds that were supplied by the mother plant [21]. During germination, triacylglycerol (TAG) is degraded via *β*-oxidation, and the released fatty acids are converted to sucrose through the glyoxylate cycle and gluconeogenesis. Thereby PEP is formed from oxaloacetate by the cytosolic PEP carboxykinase (PEPCK) [22]. Possibly, conditions favoring gluconeogenic respiration, for example high ATP levels, restrict some cPK isoforms more than others. Interestingly, cPK4 and cPK5 remained unaffected by ATP levels, while cPK1 and cPK3 were subjects of strong ATP inhibition (Figure 7). In general, gene expression is only one level of regulation and does not necessarily reflect the protein content in the respective tissue, since cPK has been reported also to be strongly regulated by protein degradation [14,23]. Furthermore, we demonstrated that full cPK activity depends on the formation of subgroup complexes (Figure 7). This leads to the possibility that a comparatively weakly expressed isoform that is part of a complex might still have an essential impact on total cPK activity. We induced the expression of cPK4 and cPK5-GUS via cold treatment, as we could not detect any expression under ordinary conditions. This result is in line with published microarray data [15]. Exposure to low temperatures enhances freezing tolerance, a versatile process involving various alterations in biochemical processes and global changes in gene expression that are accompanied by a shift in the composition of primary and secondary metabolites [24,25,26,27]. Under these conditions cell division and expansion is actively inhibited, leading to sink limitation and subsequent accumulation of carbohydrates [28]. The chilling response involves an acceleration in levels of *γ*-aminobutyric acid (GABA), proline and sucrose [29], metabolites that were shown to be involved in protecting membranes and proteins from freezing damage [30,31,32]. Under these conditions, cPKs have to maintain TCA cycle flux independent of the cytosolic ATP status, as GABA and proline depend on TCA cycle intermediates.

### 3.4. Cytosolic Pyruvate Kinases are Vital for Energy Allocation

Cytosolic PK is a key player in energy allocation, as it generates ATP in the cytosol, supplies pyruvate to the TCA cycle and thereby drives mitochondrial ATP synthesis. Cytosolic PK isoforms were strongly expressed in the leaf vasculature (Figure 6). This is expected since most cells of the vasculature are heterotrophic and depend on ATP generated via glycolysis and mitochondrial respiration. Furthermore, this enables fast access to carbohydrate substrates, for example sucrose, that is delivered from photosynthetic source tissues via the phloem sap. Our GUS experiments also revealed expression of *cPK1*, *cPK2* and *cPK3* in mesophyll cells. This expression became more pronounced at the end of the dark period, underlining the increased respiratory activity, which is normally present during the night. Furthermore, we observed distinct GUS expression in root tips, shoot apical meristems and leaf primordia (Figure 3 and Figure 4A,K–Q), indicating a significant role of cPK isoforms in ATP and pyruvate provision to these young, developing and highly energy-consuming tissues.

### 3.5. Pyruvate Kinase Enzymes are Differently Regulated by Metabolites

Several glycolytic enzymes underlie negative allosteric regulation by TCA cycle intermediates [33]. A major issue is to control the cellular NADH + H^+^/NAD^+^ and ATP/ADP ratios in order to sustain tolerable physiological conditions. This is achieved by the pH-dependent balance between citrate and isocitrate formation, which determines the overall TCA cycle flux [34]. Our kinetic characterization demonstrated that all five cytosolic PK enzymes from *Arabidopsis thaliana* are strongly inhibited in the presence of citrate, confirming previous studies on PKs [33]. cPK1 underlies a further mechanism of control because its activity was affected by aspartate and glutamate (Table 2). This might provide a feedback control balancing the generation of carbon skeletons required for NH_4_^+^ assimilation in tissues highly active in amino acid biosynthesis. Furthermore, the activity of cPK1, cPK2 and cPK3 was negatively controlled by ATP, which suggests that these isoforms are inhibited in vivo under sufficient energy supply. Activities of cPK4 and cPK5 were not affected by ATP, indicating that both isoforms act independently of the energy status of the cell, for instance under stress-related conditions. In summary, our data show that *Arabidopsis thaliana* encodes five isoenzymes with individual regulatory properties, allowing a versatile system of cPK control.

### 3.6. Cytosolic Pyruvate Kinases are Regulated by Subgroup Association and Dissociation

Most glycolytic regulatory enzymes, including PFK, PEPC and PK, were shown to exist as oligomers, whereby many of them can reversibly dissociate after binding effector molecules. This is often accompanied by an altered enzyme activity and therefore provides a mechanism for regulation [33]. Our catalytic characterization of cPK enzymes supports this hypothesis, since single isoform activities are significantly lower than activities of equimolar mixtures of specific isoforms (Figure 7). For plastidial PK enzymes, it was even reported that single enzymes or their respective homo oligomers, have no activity at all [5]. Thus, enzyme oligomerization seems to be a common mechanism to regulated PK enzyme activity.

The determined enzyme pairs are based not simply on sequence homology, but they are more in agreement with the tissue-specific expression pattern of the respective isoforms observed in our GUS studies. Accordingly, the most pronounced effect on enzyme activity was observed when the isoforms cPK4 and cPK5 were combined (Figure 7), which both appear to be strongly induced by cold treatment. The observation that cPK2 and cPK3 have positive impact on the activity of each other is underlined by co-expression data, which show that expression of *cPK2* and *cPK3* is strongly correlated (ATTEDII, version 7.1), indicating both isoforms to be active in the same metabolic pathway simultaneously. Interestingly, cPK1 appears to take over a special regulatory role because it exhibited the lowest specific activity as single enzyme (Table 1), but it was able to increase the activity of other isoforms in different combinations (Figure 7). Furthermore, cPK1 seems to underlie the most multisided metabolic control (Table 2) and appears to be most generally expressed isoform, independent of tissue and developmental stages. Finally, this supports the hypothesis of a further regulatory aspect, as *cPK1* expression might be sufficient to meet energy demand alone under certain conditions, whereas a second isoform is expressed additionally at raised energy need.

## 4. Materials and Methods

### 4.1. Assaying Tissue-Specific Pyruvate Kinase Expression by Promoter-GUS Fusion

To analyze the localization of pyruvate kinase expression during plant growth and development β-glucuronidase (GUS) fusion constructs were stably transformed into *Arabidopsis thaliana*. The first 16 days after germination were investigated on seedlings grown on half strength MS (0.5% agarose), whereas samples of later developmental stages were taken from plants grown on soil in a growth chamber. For diurnal expression analysis plants were grown under short day conditions (16 h dark, 8 h light, 160 μE·m^−2^·s^−1^). Histochemical staining of transgenic plants was performed by vacuum infiltration with staining solution (0.1M NaPO_4_ pH 7.2, 10 mM EDTA, 0.5 mM K_3_Fe[CN]_6_, 0.5 mM K_4_Fe[CN]_6_, 10% Triton x-100) supplied with 1 mM of the substrate of β-glucuronidase x-Gluc (5-bromo-4-chloro-3-indolyl *s*-d-glucuronic acid, solved in dimethylformamide) in an evacuated exsiccator according to the protocol established by Jefferson et al. (1987) [35]. For staining, the samples were incubated for 37 °C overnight and subsequently relieved from chlorophyll with 80% EtOH at 60 °C. Stained younger plants were documented with a Leica binocular (S8APO, equipped with an EC3 camera, Leica), and pictures were processed with the compatible LAS EZ imaging software (Leica). Older plants were photographed with a digital single-lens reflex camera (Sony α330). GUS-stained tissues and plants shown in this work represent the representative results of at least four independent lines for each construct.

### 4.2. Determination of Protein Localization in Nicotiana benthamiana

For transient transformation of *Nicotiana benthamiana* plants, *Agrobacterium tumefaciens* cells of the strain GV3101 pMP90 harboring the respective c-terminal YFP-tagged PK-CDS were grown overnight in a 25 mL culture (YEB, 100 ng/μL carbenicillin, 25 ng/μL gentamycin, 12 ng/μL kanamycin, 100 ng/μL rifampicin). Cells were harvested by centrifugation at 4000× *g* for 10 min and 4 °C, re-suspended in 1 mL infiltration medium (5% *w*/*v* sucrose, 0.01% *v*/*v* Silwet l-77, 2 mM MgSO_4_, 0.5% *w*/*v* glucose, 450 μM acetosyringone), and OD_600_ was adjusted to 0.4. After incubation for at least 1 h on ice, and subsequent warming to room temperature, the suspension was infiltrated at the lower side of the tobacco leaves using a 1 mL blunt end tip syringe. For determination of enzyme localization, leaf samples were taken three to four days after infiltration and analyzed by confocal laser scanning microscopy using a Zeiss LSM 700 microscope. LAS AF imaging software (Leica Application Suite Advanced Fluorescence, Leica) was used for image processing and documentation.

### 4.3. Generation of Promoter-ß-Glucuronidase Fusion Constructs

Promoter-β-glucuronidase (GUS) fusion constructs were generated in order to localize the expression of selected genes involved in growth and development of *Arabidopsis thaliana*. The 5′-flanking regions of cytosolic PK genes were cloned into the gateway binary vector pGWB3, which mediates GUS fusion [36]. Initially, ~1800 bp of the promoter regions were amplified from *Arabidopsis thaliana* genomic DNA by preparative PCR and introduced into gateway pENTR vectors. For the cPK1 promoter, the *Nco*I site was inserted into the 5′ end forward primer prom_cPK1_*Nco*I_for and the *Xma*I site into the 3′ end reverse primer prom_cPK1_*Xma*I_rev to facilitate T4-ligase (New England Biolabs)-mediated ligation into pENTR4 (Invitrogen, Carslbad, USA). Oligonucleotide sequences for cloning are provided in the Appendix (Appendix Table A1).

All other constructs were generated by TOPO cloning into the pENTR/D-TOPO vector (Invitrogen) according to the manufacturer’s instructions using the following primer pairs for DNA amplification: for cPK2, prom_cPK2_TOPO_for and prom_cPK2_TOPO_rev; for cPK3, prom_cPK3_TOPO_for and prom_cPK3_TOPO_rev; for cPK4, prom_cPK4_TOPO_for and prom_cPK4_TOPO_rev; for cPK5, prom_cPK5_TOPO_for and prom_cPK5_TOPO_rev. The resulting pENTR constructs were transformed into the *E. coli* strain DH5α. After transformation, the *E. coli* were plated onto LB agar containing the respective antibiotics in order to select for successfully transformed colonies. Transformed colonies were confirmed via PCR and restriction digestion of isolated plasmid DNA as well as double-strand sequencing. The promoter fragments were cloned into the vector pGWB3 by LR reaction (LR-clonase, Invitrogen, Carslbad, CA, USA). The final plasmids were stably introduced into *Arabidopsis thaliana* plants by *Agrobacterium tumefaciens* (*A. tumefaciens*)-mediated transformation, and transformants were isolated following selection with the respective antibiotics on half-strength MS agar (Duchefa, 50 μg/mL kanamycin, 50 μg/mL hygromycin, 0.5% agarose).

### 4.4. Generation of 5x His-Tagged Fusion Constructs

For heterologous expression of cytosolic PK proteins in *E. coli*, full-length cDNA was cloned into the pET16b vector (Novagen, Darmstadt, Germany) mediating N-terminal His-tag fusion. The cDNA of cPK2 and cPK3 was amplified with the following oligonucleotides carrying the restriction enzyme recognition sites for subsequent classical ligation into the destination vector for cPK2 cPK2_*Nde*I_for and cPK2_*Nde*I_rev, for cPK3 cPK3_*Nde*I_for and cPK3_*BamH*I_rev. In order to increase the DNA yield of cPK1, cPK4 and cPK5 amplicons, the cDNA was subcloned into the pENTR/D-TOPO vector (Invitrogen, Carslbad, USA) by TOPO cloning using primers cPK1_TOPO_for and cPK1_TOPO_rev for cPK1, cPK4_TOPO_for and cPK4_TOPO_rev for cPK4, and cPK5_TOPO_for and cPK5_TOPO_rev for cPK5. Oligonucleotide sequences for cloning are provided in the Appendix (Appendix Table A1). Amplified cDNA was digested with the indicated enzymes, purified by gel extraction and cloned into pET-16b by T4 Ligase (New England Biolabs, Ipswich, USA). Obtained plasmids were transformed into the *E. coli* strain BLR21, and the constructs were verified by colony PCR and restriction digestion.

### 4.5. Protein Purification by Metal Chelate Affinity Chromatography

After harvest and cell lysis, heterologously expressed 5xHis-PK proteins were purified by Ni-NTA affinity chromatography in a batch procedure. The cleared lysate was mixed for 1 h at 4 °C together with 2 mL Ni-NTA agarose beads (Macherey Nagel, Düren, Germany), which had been washed earlier with lysis buffer (50 mM TRIS-HCl pH 8, 300 mM NaCl, 1 mM imidazole). The supernatant obtained by centrifugation at 300× *g* for 5 minutes at 4 °C was removed. Afterwards, the Ni-NTA beads were resuspended in 4 mL wash buffer (50 mM TRIS-HCl pH 8, 300 mM NaCl, 2 mM imidazole) and added to a self-made column plugged with cotton wool. The flow through was collected and a further washing step followed. Matrix-bound proteins were eluted stepwise by application of four times 0.5 mL elution buffer (50 mM TRIS-HCl pH 8, 300 mM NaCl, 250 mM imidazole). The obtained elution fractions were desalted afterwards.

### 4.6. Desalting of Purified Protein by Size Exclusion Chromatography

After Ni-NTA chromatography, obtained elution fractions were desalted on Sephadex G-25 columns (NAP-5, GE-Healthcare). Columns were equilibrated 3 times with 2 mL PK storage buffer (100 mM TRIS-HCl pH 8, 1 mM dithiothreitol, 1 mM EDTA, 5 mM MgCl_2_) before the elution fractions were added and PK protein was eluted in 1 mL PK storage buffer. Desalted protein was stored on ice until protein quantification, SDS-page (Appendix Figure A4) and kinetic characterization studies (Appendix Figure A5) followed.

### 4.7. Kinetic Characterization of Pyruvate Kinases

The activity of purified cPK isoenzymes was assayed in a coupled reaction system involving lactate dehydrogenase (LDH) according to a previously described method by Plaxton [19], based on photometric detection of NAD+ formation following LDH-mediated NADH oxidation. PK activity measurement was performed in a total volume of 200 μL reaction solution (50 mM TRIS-HCl, pH 7.5, 50 mM KCl, 10 mM MgCl_2_, 5% *w*/*v* PEG 200, 1 mM DTT, 0.15 mM NADH) containing LDH (20 U/mL LDH, Roche) applying 5-10 μL of freshly purified PK protein in diverse concentrations. The shift in absorption was detected at ʎ = 340 nm in a 96-well format in a microplate reader (Infinite M200, Tecan, Männedorf, Schweiz) at 25 °C. To start reactions, substrates and potential effectors of PK enzymes were added to all reaction batches simultaneously applying a house made applicator with 96 spatulas.

## Figures and Tables

**Table 1:** In vitro catalytic properties of cPK enzymes heterologously expressed in *Escherichia coli*: Michaelis constant *Km*, enzymes maximum rate *Vmax* and turnover number *kcat*.

Isoform	*Vmax* (U/mg)	*kcat* (1/s)	*Km* (mM)	
			ADP	PEP
**cPK1**	1.4	1.53	0.15	0.06
**cPK2**	2.8	3.05	0.03	0.76
**cPK3**	2.2	2.4	0.14	0.17
**cPK4**	3.9	4.0	0.07	0.17
**cPK5**	6.4	6.68	0.34	0.05

**Table 2:** Allosteric effects on cPK enzyme activity after application of F1.6BP (1 mM), serine (0.2 mM), AMP (0.1 mM), ATP (2 mM), glutamate (0.2 mM), aspartate (0.2 mM) and citrate (4 mM) compared to control.

Isoform	F1.6BP	Serine	AMP	ATP	Glutamate	Aspartate	Citrate
**cPK1**	122%	99%	102%	7%	85%	69%	17%
**cPK2**	104%	112%	90%	76%	99%	97%	26%
**cPK3**	101%	99%	109%	27%	102%	94%	7%
**cPK4**	104%	100%	97%	84%	100%	97%	3%
**cPK5**	97%	101%	97%	95%	100%	98%	2%

## Appendix A

**Table A1:** Oligonucleotide sequences for cloning.

Name	Sequence
prom_cPK1_*Nco*I_for	ggccatggaatgaatgtttttgcagtat
prom_cPK1_*Xma*I_rev	ccccgggttttttctccttctcaagtt
prom_cPK2_TOPO_for	cacctactatcagctattagaattatatatc
prom_cPK2_TOPO_rev	cgctaagtgagaaaaaaaacag
prom_cPK3_TOPO_for	caccctctgtgatggatccaagtag
prom_cPK3_TOPO_rev	gtaaatctccaaaaacctaatc
prom_cPK4_TOPO_for	caccatttaccgaagtgggttagatcgg
prom_cPK4_TOPO_rev	agttgctgatcagaattcggaga
prom_cPK5_TOPO_for	cacctccttctagttttacgaaca
prom_cPK5_TOPO_rev	cttcggtgacggaagggagagagatc
cPK2_*Nde*I_for	gggcatatgatggcgatgatagagcaaagg
cPK2_*Nde*I_rev	ccccatatgtcacttgacggtcaagatcttgatca
cPK3_*Nde*I_for	gggcatatgatgtcgaacatagacatagaag
cPK3_*BamH*I_rev	cccggatcctacttcaccacacagatcttg
cPK1_TOPO_for	caccacccatatgatgtcgaacatagacatagaaggg
cPK1_TOPO_rev	ggtcatatgtcacttaaccacacagatcttaa
cPK4_TOPO_for	caccaaccatatgatgcattcaagtcatctcc
cPK4_TOPO_rev	aacggatccctaatcctctagctcgatgattt
cPK5_TOPO_for	caccaaccatatgatgcattccagtcatcttct
cPK5_TOPO_rev	gttggatccttaatcctcaagctcaatga
